# A Thermoplastic Multilayered Carbon-Fabric/Polycarbonate Laminate Prepared by a Two-Step Hot-Press Technique

**DOI:** 10.3390/polym10070720

**Published:** 2018-06-30

**Authors:** Xiaokang Liu, Binbin Yang, Longsheng Lu, Zhenping Wan, Yong Tang

**Affiliations:** School of Mechanical & Automotive Engineering, South China University of Technology, 381# Wushan Road, Guangzhou 510641, China; liuxk58@scut.edu.cn (X.L.); yangbb922@163.com (B.Y.); zhpwan@scut.edu.cn (Z.W.); ytang@scut.edu.cn (Y.T.)

**Keywords:** laminate, carbon fiber, flexural test, tensile test, impact test, composite

## Abstract

Carbon fiber (CF) reinforced thermoplastic composites have gradually become increasingly popular in composite production owing to their lower hazard level, good structural flexibility and recyclability. In this work, a multilayered carbon–fabric/polycarbonate laminate (multi-CFPL) was fabricated by a two-step hot-press process, mainly based on the thermoplastic properties of its polycarbonate (PC) matrix. Different from the conventional one-step method, the two-step hot-press process was composed of two separate procedures. First, a unit-hot-press operation was introduced to prepare a single-layered carbon–fabric/PC laminate (simplified as unit-CFPL). Subsequently, a laminating-hot-press was employed to compress several as-prepared unit-CFPLs bonded together. This combined process aims to reduce the hot-press temperature and pressure, as well as facilitate the structure designability of this new composite. Several mechanical investigations were conducted to analyze the effect of the hot-press parameters and unit-CFPL numbers on the performance of this multi-CFPL material, including flexural, uniaxial tensile and impact tests. The results reveal that the multi-CFPL exhibits a good stability of flexural and tensile properties in terms of strength and modulus. Furthermore, during impact tests, the multi-CFPL presents an accelerated growth of peak force and energy absorption capability with increasing unit-CFPL layers.

## 1. Introduction

Carbon fiber reinforced plastic (CFRP) laminates contain several layers of unit composites bonded together to form a united body. Compared with conventional homogeneous composites, CFRP laminates offer many superior advantages due to their remarkable specific strength and modulus [1], corrosion resistance, fatigue properties [2] and impact resistance. Therefore, the CFRP laminates are widely applied in industries such as wind turbine blades in clean energy fields [3] and fuselages, wing skins, and other critical components in aircraft fields [4,5]. In addition, the CFRP laminates present highly flexible designability [6,7,8,9], making their fabrication process easily tailored to meet different application requirements.

At present, the most popular CFRP laminates are thermosetting, and their most common matrices are epoxy [10]. Moreover, some additional chemical additives may be incorporated into the epoxy to achieve special performance such as high toughness [11] and self-healing during abrasion damage [12]. Unfortunately, these additives are always harmful to the environment and human beings. To achieve a green manufacturing process, researchers are attempting to increase the structural flexibility and recyclability of CFRP laminates [13]. However, it is difficult to realize the recycling of epoxy-based CFRP laminates because of their inherent thermal setting attributes. Therefore, another CFRP laminate candidate, carbon fiber (CF) reinforced thermoplastic laminate, has gradually become increasingly popular in composite production owing to their less hazardous chemical composition and high productivity production potential [14,15].

Traditionally, a film/fabric stacking technique followed by a hot-press process is a convenient and effective method to prepare high-performance CF-reinforced thermoplastic laminates [13,15,16]. In this method, all raw materials placed in designated positions, layer-by-layer, are directly hot-pressed to obtain a united body in one operation, which, in this work, is defined as one-step hot-press technique. Sufficient CF-matrix impregnation [2] and precise CF position [7] are two key points to be addressed in processing because they significantly affect the mechanical properties of the laminates. To achieve sufficient impregnation, high molding pressure and temperature are always adopted to enhance the flowability of the matrix during hot-press processing. Ozaki et al. [17] analyzed the effect of the hot-press parameters on the bending properties of a CF/polycarbonate (PC) laminate, and Tanaka et al. [18] investigated the interfacial bonding strength and the interlaminar shear strength of this laminate. They suggested that an optimal hot-press temperature should be set at approximately 300 °C and 6 MPa pressure accompanied by sufficient holding time (half an hour of whole hot-press process or several minutes of maximum temperature) to obtain secure CF-PC interfacial bonding. Qian et al. [19] researched the effect of polycarbonate film surface morphology and oxygen plasma treatment on mechanical properties of composite laminates. They selected relatively low temperature at 180 °C to fabricate laminates, which at cost of 2 h of hot-press time. Even in the fabrication of carbon nanotube/PC composite, Choi et al. [20] still set melting temperature of twin extruder at 300 °C.

Unfortunately, a critical shortcoming has emerged under high pressure and temperature during laminate preparation. Under high pressure and temperature molding situations, the melted matrix can easily scour CF tows away from their pre-placed positions, thereby causing the mechanical performance of the laminates to deteriorate [7,21]. To address this problem, on the one hand, researchers have tried to increase the CF density to enhance the rigidity of the fabric to resist the fluid scouring force. However, high CF density undoubtedly requires further growth of the molding pressure and temperature to guarantee sufficient CF-matrix impregnation. On the other hand, the requirements of excessive pressure and temperature can be relieved by reducing the CF density of the woven fabric, which can be achieved by using spread CF tows to weave into the fabrics. For example, El-Dessouky and Lawrence [22] successfully adapted spread CF tows to make high-performance thermoplastic laminates because of the improvement of the CF-matrix impregnation process. By applying spread CF tows, previous work [23] successfully manufactured an ultra-thin (thickness ≤0.3 mm) single-layered carbon-fabric/PC laminate (simplified as unit-CFPL) under relatively low molding parameters—240 °C molding temperature, 6 MPa molding pressure and 3.5 min molding time. These parameters were much lower than the parameters that were necessary without spread CF tows [17,18]. Thus, it is promising to make multilayered laminates by using spread CF tows as raw material.

For a multilayered laminate fabricated in a one-step hot-press technique under certain conditions, the CF-matrix interfacial bonding strength will significantly decrease as its layer number or thickness increases because of its significant impregnation resistance gradient in the direction of its thickness. Normally influenced by the temperature gradient and thermal conductivity, different layers in thick multilayered laminates must present different impregnation rates, i.e., outer layers will complete a suitable impregnation process with lower molding parameters than the inner layers. Nevertheless, slightly exceeding the temperature and holding time would be able to displace spread CF tows from their predetermined position for each layer because of their bad fiber collection [23]. Therefore, it is improper to fabricate multilayered laminates using a one-step hot-press technology because it is very difficult to ensure both CF-matrix impregnation and correct fiber position of each layer.

As Ramalakshmi et al. [24] illustrated, in addition to affording loads, the matrix also played a role of holding the fiber phase in the desired position. Therefore, a separate process called a two-step hot-press technology has been identified and takes advantage of the reprocessing property of thermoplastic resin. This technique consists of unit-hot-press and laminating-hot-press in sequence, which is a quick and precise way that ensures both the CF tow position and impregnation. In addition, the fabrication parameters can also be optimized more precisely layer-by-layer.

In this work, a multi-CFPL was fabricated by a two-step hot-press method. Although there are many studies focused on the mechanical properties of CFRP laminates, little attention has been paid to the laminates produced repeatedly by the same composite material. The purpose of this work was to investigate the mechanical properties of multi-CFPLs made by the two-step hot-press technique and to certify the reliability of this method. For performance testing, the flexural failure is one of the most important mechanical properties in the application to blades [3] and aircraft wings [5]. Additionally, impact damage is commonly recognized as the greatest failure threat to laminated composites [25,26]. Therefore, a set of different multi-CFPLs were prepared and studied to test the flexural, tensile and impact properties. Limited by experimental amounts, the layer numbers of laminates were no more than 10, with an appropriate thickness interval (≤2.3 mm thick) in most studies [27,28,29].

## 2. Experimental Procedures

### 2.1. Multi-CFPL Preparation

The raw materials of a multi-CFPL consist of polyacrylonitrile (PAN)-based CFs and PC, with CFs used as reinforcement and PC serving as the matrix.

The CF was T700SC (12 K, Toray Industries, Tokyo, Japan) in a continuous tow shape. Its major physical properties were 4.9 GPa tensile strength and 230 GPa tensile modulus [30]. Its overall geometric dimensions were 20 mm in width and 0.040 mm in thickness, obtained by a further pneumatic spreading technique [31]. The PC film, purchased from LEXAN SABIC Company, Saudi Arabia, was 0.125 ± 0.002 mm in thickness, 65 MPa in tensile strength, 2506 MPa in tensile modulus, and 153 °C in glass transition temperature, which was measured by a differential scanning calorimeter (Model 214, NETZSCH, Selb, Germany). No further processing was applied.

**Figure 1:**
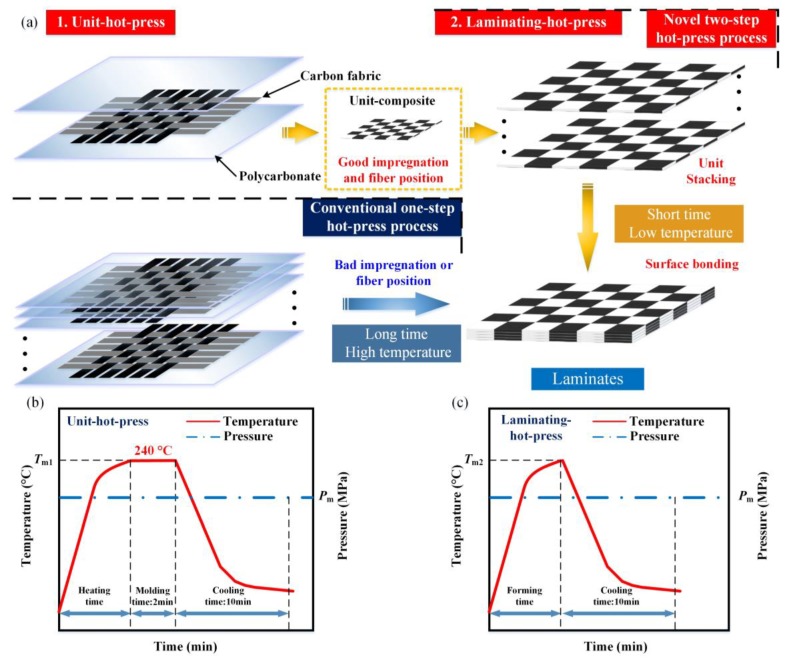
The schematic diagram of multi-CFPL fabrication using the two-step hot-press process: (**a**) Main operation step illustration; (**b**) the molding history of the unit-hot-press, in which *T*_m1_ is the hot-press temperature and *P*_m_ is the hot-press pressure; (**c**) the molding history of the laminating-hot-press, in which *T*_m2_ is the forming temperature.

The multi-CFPL was prepared by a two-step hot-press technique, i.e., a unit-hot-press operation followed by a laminating-hot-press process, as shown in Figure 1a. At first, the unit-hot-press operation was introduced to prepare unit-CFPLs. Based on a series of pre-experiments [23], the processing parameters of the unit-hot-press were optimized as 240 °C molding temperature, 6 MPa molding pressure and 2 min holding time. This holding time was set at nearly half of the optimal value (3.5 min) that was determined in previous work [23] based on the consideration of making a reservation for following the laminating-hot-press. A detailed molding history is shown in Figure 1b, and the specific fabrication information can be found in previous work [23].

After the unit-hot-press operation, the laminating-hot-press was employed to compress as-prepared unit-CFPLs bonded together. All unit-CFPLs were first cut into the same dimensions of 120 mm × 120 mm. Then, several layers of unit-CFPL were piled up in an orderly manner in a mold with two release films on each outside surface. Finally, a multi-CFPL was compressed together and finished by an additional laminating-hot-press. Because the interfacial bonding between CFs and PC had been achieved well by the unit-hot-press, the laminating-hot-press could be carried out at a relatively lower temperature and with a shorter holding time, as shown in Figure 1c. The detailed hot-press parameters of the laminating-hot-press will be determined via a series of experiments.

### 2.2. Flexural and Uniaxial Tensile Testing

The flexural and tensile properties are regarded as fundamental characteristics when a laminated composite is used as the structural material [17]. It should be noted that some multi-CFPLs were also attempted to manufacture by the one-step hot-press technique, using similar hot-press parameters as previous work [23]. However, the laminates presented very bad fiber orientation which were not viable for comparison.

**Figure 2:**
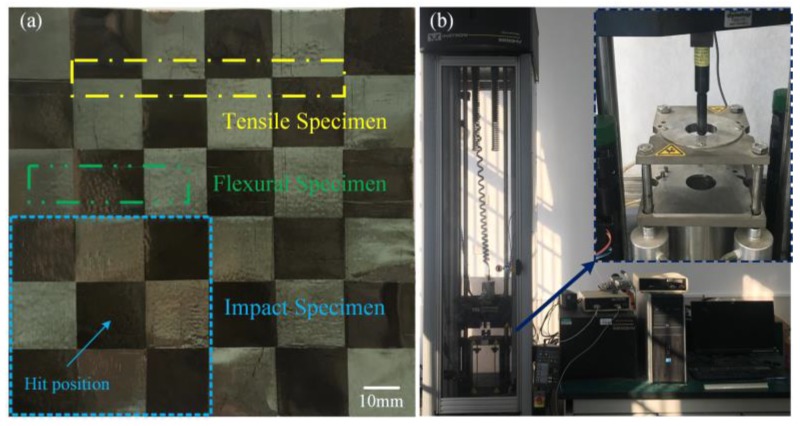
Preparation of specimens and testing apparatus: (**a**) illustration of the sampling position for the tensile, flexural and impact tests; (**b**) the scene photos of a drop-weight impact test.

To evaluate the effect of the hot-press parameters and unit-CFPL layer numbers on the flexural behavior of multi-CFPLs, flexural tests were conducted on a universal material testing machine (Model Z100, Zwick/Roell, Ulm, Germany) equipped with a 1 kN load based on the testing standard of ASTM D790 [32]. Various multi-CFPLs with 7 to 10 layers of unit-CFPLs have a shape of 1.60 mm to 2.28 mm in thickness, 12.7 mm in width and 50.8 mm in length. All specimens were precisely cut from as-tested laminates by a CNC milling machine (Guangzhou Machine Tool Works Co., Ltd., Guangzhou, China). Subsequently, the specimens were tested at a cross-head speed of 0.01 mm/mm/min. A span-to-depth ratio of 16 was set between two specimen supports [28]. Their selected positions are shown as the green double-dot-dashed outline exhibited in Figure 2a. In addition, a series of uniaxial tensile tests were also conducted on the same testing apparatus at the speed of 2 mm/min using specimens with 10 mm × 80 mm dimensions, according to the ASTM Standard D 3039 [33]. All tensile specimens were extracted from as-tested multi-CFPLs at the position illustrated as the yellow dot-dashed outline displayed in Figure 2a. In addition, all tensile specimens were end-tabbed using epoxy resin to prevent any potential failures occurring at the specimen/grip interface. Limited by the relatively poor adhesion ability between the epoxy tab and the specimens, the tensile tests could only be successfully conducted from the 1- to 4-layer laminates in this work. To minimize testing errors, all tests were carried out with five replicates for each parameter.

### 2.3. Impact Testing

The impact resistance of multi-CFPLs was tested following the ASTM Standard D 7136 [34], using a drop-weight impact machine (9250 HV, Instron, Norwood, MA, USA). Each impact test was performed by dropping a 12.7 mm-diameter hemispherical striker with a 17.6055 kg mass on a specimen mounted in a pneumatic fixture. Subsequently, the corresponding dynamic response of the specimen was recorded and analyzed. Each impact specimen was tailored to the dimensions of 60 mm × 60 mm, which was large enough to be completely clamped in the fixture as the blue-dashed outline shown in Figure 2a. Other testing conditions included 800 kHz sampling rate, 15 J applied energy, and 86.9 mm drop height. To eliminate potential testing errors caused by strike position difference, all impact specimens were penetrated in the middle of the CF tows rather than the edge [34].

### 2.4. Other Characterizations

To evaluate the interfacial adhesion behavior between CF filaments and PC polymers, the cross-sections and failure sections of multi-CFPLs selected after the testing were observed via a digital optical microscope (VHX-1000, Keyence Company, Osaka, Japan).

## 3. Results and Discussion

### 3.1. The Parameter Selection for the Laminating-Hot-Press Process

The performance of a multi-CFPL is greatly affected by its molding parameters [35]. In previous work [23], the molding parameters of the unit-hot-press have been thoroughly investigated. Therefore, in this work, a series of experiments were conducted to optimize the processing parameters of the laminating-hot-press. Based on the experience of the unit-hot-press, the forming temperature of the laminating-hot-press was pre-set, varying from 190 to 230 °C in increments of 10 °C to elucidate the effect of temperature, while the forming pressure was maintained at 6 MPa, the forming time at three min and the cooling time at 10 min. Moreover, in these experiments, all multi-CFPLs were made from a seven-layer unit-CFPL, with a finished thickness (1.6 mm) that was appropriate for flexural tests.

**Figure 3:**
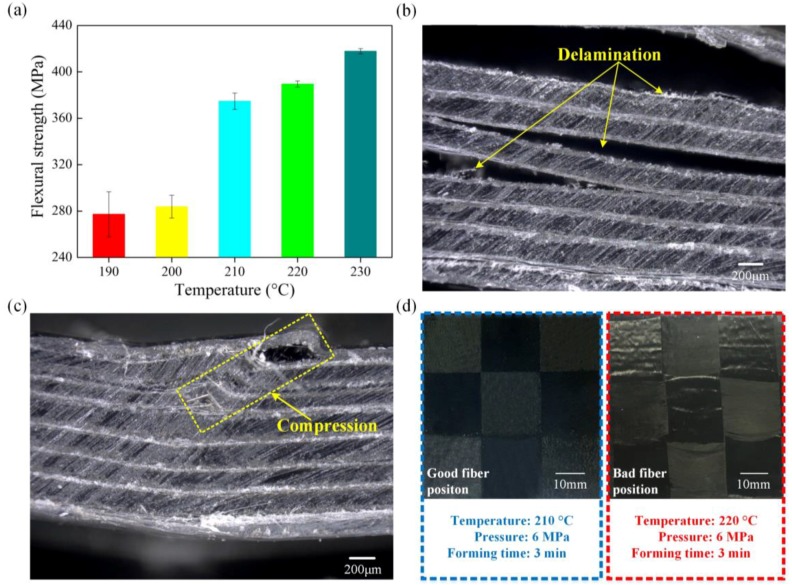
The flexural properties of 7-layer multi-CFPLs fabricated by different forming temperatures in the laminating-hot-press: (**a**) flexural strength bars; (**b**,**c**) are the digital pictures of multi-CFPLs prepared at 200 °C and 210 °C forming temperature, respectively; (**d**) shows the visual image comparison between the multi-CFPL prepared at 210 °C and 220 °C forming temperature.

Figure 3 shows the flexural properties of multi-CFPLs prepared by different forming temperatures in the laminating-hot-press. The results indicate that the flexural strength increases with increasing forming temperature. As the forming temperature increases from 190 to 230 °C, the flexural strength increases from 277.22 to 417.89 MPa. However, there is a rapid growth gap in this work between 200 and 210 °C, which divides the growth rate of flexural strength into two parts. This variation tendency can be explained using two typical failure images illustrated as the insets in Figure 3. When the forming temperature is below 210 °C, the as-prepared multi-CFPLs show several obvious delamination failure areas within each unit-CFPL layer, indicating insufficient interfacial bonding strength. This defect is similar to the interlaminar shear mode described as the Standard ASTM D2344 [36]. When the forming temperature is above 210 °C, the failure image of the multi-CFPL looks identical to the failure image caused by compression failure [37] owing to exceeding the compression limit of the material. No obvious delamination trails were found at this temperature, implying sufficient interfacial bonding strength among each of the unit-CFPLs. Therefore, the increase in the forming temperature in the laminating-hot-press can promote the impregnation between CF and PC and transfer the failure from interlaminar shear to compression fracture, thereby significantly enhancing the flexural ability of the laminates. However, there is a maximum limitation on the forming temperature. An excessively high forming temperature will generate an extremely high scouring force in the melting matrix and induce quality defects. As shown in Figure 3d, the CF location and the pre-determined uniformity associated with the weaving process are easily disturbed by a high scouring force, thereby reducing the stability of the quality of the laminated composites [21]. Under a comprehensive consideration, a set of optimal laminating parameters for this work was selected at 210 °C forming temperature and 6 MPa forming pressure.

### 3.2. Morphology Observation

**Figure 4:**
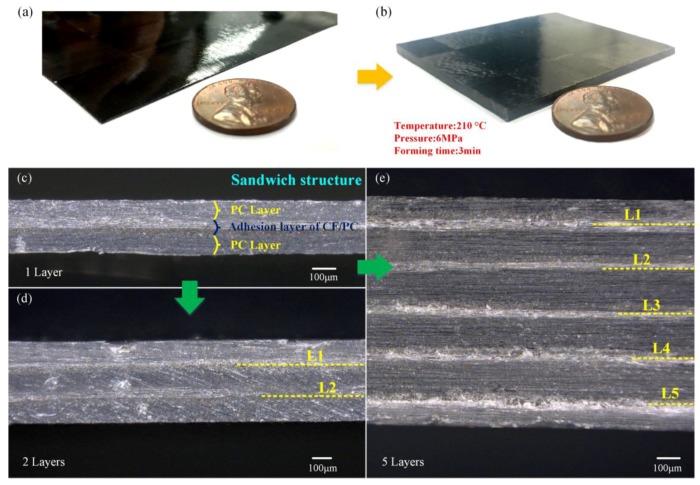
The morphologies of a unit-CFPL and several multi-CFPLs prepared by optimal laminating parameters: (**a**,**b**) show the visual images of 1-layer and 10-layer multi-CFPLs from the axonometric view, respectively; (**c**–**e**) shows the cross sections of 1-layer, 2-layer and 5-layer multi-CFPLs, respectively.

With the optimal unit-hot-press and laminating-hot-press parameters mentioned above, a unit-CFPL and a 10-layer multi-CFPL were manufactured for comparative observation, as shown in Figure 4a,b, respectively. From the appearance, the surfaces of both the unit-CFPL and the multi-CFPL are smooth and flat without any obvious bubbles, and a good CF uniform distribution is displayed, i.e., all weave CF tows in these specimens are stuck at their pre-designed positions. In addition, it is interesting to determine that the thickness of the multi-CFPL is slightly lower than the layer number times the thickness of its identical unit-CFPL. For example, the unit-CFPL is 0.26 mm-thick, and the as-prepared 10-layer multi-CFPL is 2.28 mm-thick, which is smaller than 10 mm × 0.26 mm. This thickness reduction is attributed to two reasons. One reason is that some PC has further pressed into the pores of the weave carbon fabric by laminating-hot-press, and another reason is that some PC is squeezed out. Therefore, the thickness reduction can be used as an indirect indication of enhancing the interfacial bonding strength by laminating-hot-press.

To evaluate the impregnation quality of the CF-matrix and the interfacial bonding quality of the layer–layer, several multi-CFPLs with 1, 2 and 5 layers of unit-CFPL were carefully cut to observe their cross-sections, as shown in Figure 4c–e. All sections exhibit a good bonding interface, i.e., all unit-CFPLs combine well with each other after laminating-hot-press. In addition, there is no visible void, bubble or delamination inside each unit-CFPL. As claimed by Moaseri et al. [38], a good combination of carbon fabric and PC films suggested outstanding mechanical properties. Moreover, there are clear boundary lines between the weave carbon fabric and PC films due to their substantially different material compositions. These lines are straight and tidy as well as parallel to each other, similar to the pre-arranged unit-CFPL layers. This appearance can be used as evidence that the major properties of unit-CFPLs may be reserved in their as-prepared multi-CFPLs.

### 3.3. Flexural Analysis

Generally, the impact property of a component is closely related to its flexural properties [26]. Therefore, the flexural behavior of the multi-CFPLs was first analyzed to help in the understanding of impact behaviors. Due to the limitation of the testing standard ASTM D790 [32], a basic thickness of 1.6 mm for the specimen is required. Therefore, all flexural tests in this work were conducted on the multi-CFPLs consisting of 7 to 10 layers of unit-CFPLs, with thicknesses larger than 1.6 mm.

**Figure 5:**
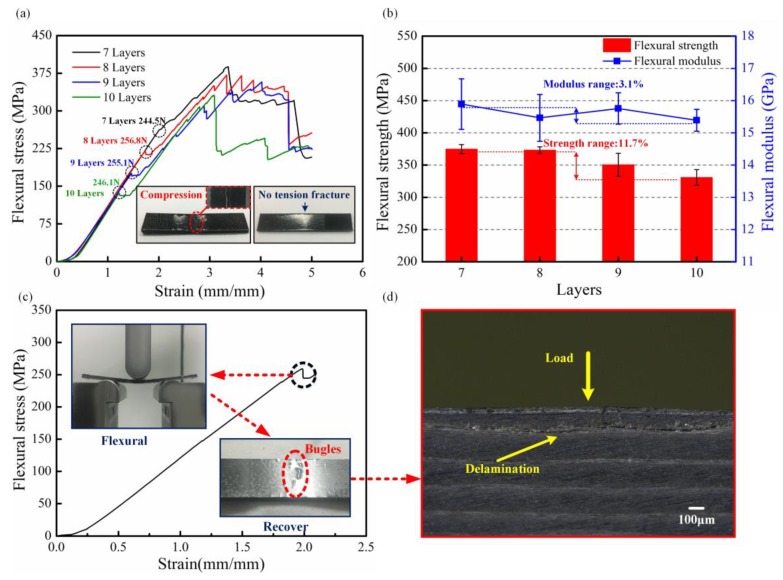
Flexural properties of multi-CFPL with different unit-CFPL numbers: (**a**) Stress-strain curves; (**b**) flexural strength bars and modulus curves; (**c**,**d**) are the testing curve and observation of the first failure point in the flexural stress curve done on the CFPL with a 7-layer unit-CFPL.

Figure 5a exhibits the strain-stress curve of multi-CFPLs. All these strain-stress curves display a similar developing tendency. In the initial stage, the flexural stress shows a nonlinear growth over the strain when the strain is below 0.3 mm/mm. In this stage, the cross-head of the testing machine comes in contact with the upper surface of the specimen. Because there are many pure PC layers in the inner structure, the multi-CFPL will easily appear to have a certain retreat under the elastic deformation of the PC. As the strain increases further, the flexural stress will exhibit a linear rising period until the first failure point appears (as the dash circles show in Figure 5a). This linear rising period is known as the Hookean region [32], and the entire laminate begins to bear bending stress. The slopes of each curve are almost coincident, and their first failure points are arranged almost equidistantly along with the reduction of the layer numbers. Next, the flexural stress has a slight reduction and holds a value for a brief time. Finally, the stress continues to rise and comes to a severe damage stage, and the multi-CFPL presents a ductile flexural failure other than a catastrophic break [8]. When the damage is just beginning to appear in a multi-CFPL, the PC layers could act as protectors to avoid further cracking because of their good plasticity. Therefore, the final stage would exhibit many slight reduction points rather than an instant fracture such as brittle rupture. This phenomenon is ideal for the actual application of composites.

Figure 5b shows the flexural strengths and modulus of multi-CFPLs versus the unit-CFPL layer. In general, the flexural strength presents a slight tendency to decline as the layers of the unit-CFPL increase. For instance, the flexural strength decreases from 374.78 to 330.75 MPa as the layer numbers increase from 7 to 10. Katsiropoulos et al. [16] reported that with increasing numbers of layers in a laminate, some bond line defects (e.g., voids, kissing bonds, and porosity) were increased, adversely affecting the mechanical properties of bonding joints. Moreover, in a flexural test, the bending moment and the shear force exist simultaneously, resulting in a mixing failure mode including delamination, tension and compression [39]. Therefore, the flexural strength of a multi-CFPL is hard to keep stable with the growth of the unit-CFPL layers. However, the flexural modulus of a multi-CFPL is relatively stable. As the layer number increases from 7 to 10, the flexural modulus fluctuates slightly up and down between 15.39 to 15.89 GPa, which demonstrates the stability of the rigidity of this laminate. This behavior is also reflected in the Hookean region of stress–strain curves.

For a further comparison, the loading forces of first failure points in the flexural stress-strain curves were calculated as given in Figure 5a. All these forces are approximately 250 N, demonstrating that the first failure force may be a specific property of this two-step hot-press laminate and has nothing to do with the unit-CFPL layer numbers. To validate this induced conclusion, an additional flexural test was conducted on a seven-layer multi-CFPL. In this test, the cross-head was immediately stopped and slowly removed from the laminate when its first failure point in the stress curves emerged, as shown in Figure 5c. The result indicated that the bending deformation of the multi-CFPL would gradually be recovered when its flexural load was removed, which is similar to the flexural behavior of an elastic beam. Therefore, the multi-CFPL at this moment is still treated in the elastic deformation. However, some bulges were observed on the upper surface layer of multi-CFPL. For a deeper understanding of these bulges, a partial enlarged image in cross section was acquired, as shown in Figure 5d. Some obvious defects only appeared in the first layer of the multi-CFPL. Meanwhile, there was no visible damage generated in other layers. Because there was a thick pure PC part on the upper layer which was not reinforced by carbon fabric. In addition, it was not constrained by carbon fabric as other layers either. Therefore, when the load increased to a certain degree, the surface of laminates was kinked firstly, thereby generating a similar failure force for various multi-CFPLs with different layer numbers.

**Figure 6:**
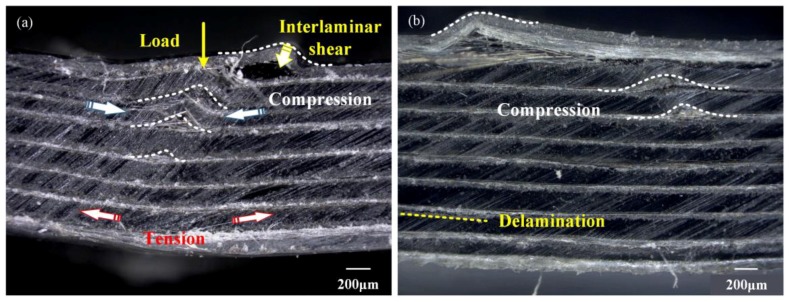

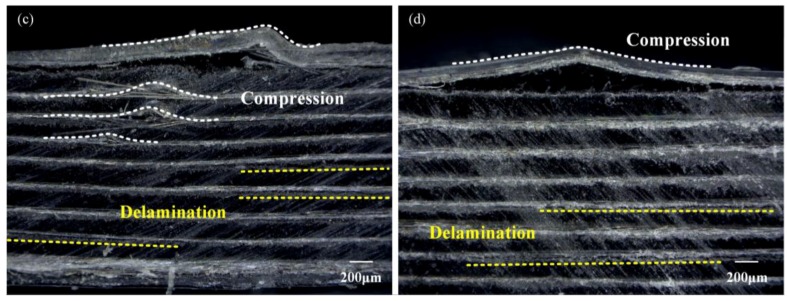
The partial enlarged images of multi-CFPLs in cross section with different layers of unit-CFPLs: (**a**) 7 layers; (**b**) 8 layers; (**c**) 9 layers; (**d**) 10 layers. All these images were taken at major failure locations after the flexural tests.

When the loading flexural stress is over its peak value, the as-tested laminates would be damaged. To determine the failure mechanism, all these failure multi-CFPLs were investigated by partial enlarged images observed in cross-section, as illustrated in Figure 6. The results indicate that these failure multi-CFPLs are dominated by compression and delamination failure modes, while tension breakage seldom occurs. Generally, the bottom layer of a multi-CFPL that is opposite the cross-head behaves under tensile stress while the upper layer suffers compressive stress during the flexural loading process [5,40]. Moreover, shear stress exists in the combination boundary between each carbon fabric and PC film. Under such a complex stress field, the fracture of multi-CFPLs will exhibit a different dominating failure mechanism with increasing layer numbers. For the 7-layer multi-CFPL, the compression mode dominates, so there are few delamination trails between the carbon fabric and the PC. When the layers increase to 10, the extent of the compression of the upper layer in multi-CFPLs gradually decreases, and its compression fracture trails decrease correspondingly, whereas the delamination defects increase and become the major part because of the increase in their shear stress. In summary, all the partial enlarged images validate the homogeneous structure of multi-CFPLs fabricated by the two-step hot-press technique.

### 3.4. Uniaxial Tensile Analysis

**Figure 7:**
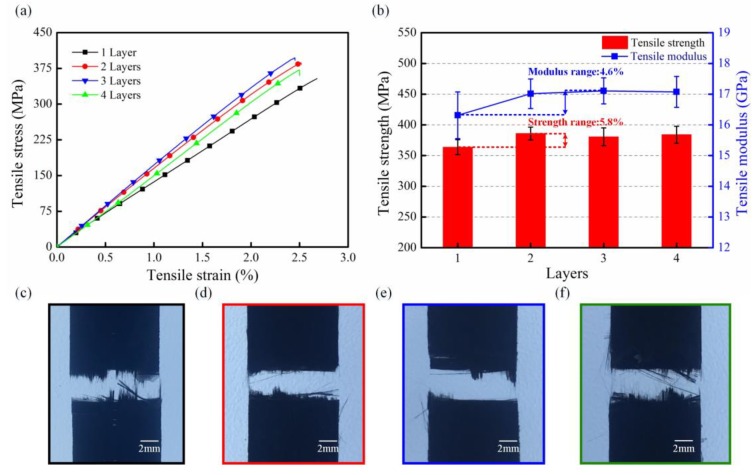
Tensile properties of the carbon–fabric/PC laminates with different layer numbers: (**a**) stress-strain curves; (**b**) tensile strength curves and tensile fracture morphologies; (**c**–**f**) are the fracture morphologies of multi-CFPLs with 1-, 2-, 3- and 4-layer unit-CFPLs, respectively.

Figure 7 illustrates the tensile properties of several multi-CFPLs with 2- to 4-layer unit-CFPLs in terms of their stress-strain responses, strengths and failure morphologies. In addition, a pristine unit-composite is set as a comparison. As shown in Figure 7a, all tensile specimens exhibit a specific linear-elastic period followed by a dramatic fracture without a yield stage, which is a classical tensile behavior of the CF-reinforced plastic composites with a high modulus [33]. Moreover, the elongations of all these multi-CFPLs are nearly equal, and their values are fluctuating around at 2.5%, while the unit-composite has a slightly higher elongation at 2.67%. For the tensile strength, all these multi-CFPLs appear to have an approximately constant value of approximately 380 MPa, which is slightly higher than the value of the unit-CFPL (360 MPa). This similar comparative result can also be observed in the aspect of the tensile modulus. The results indicate that the tensile modulus of all multi-CFPLs is as high as approximately 17 GPa, which is higher than the tensile modulus of the unit-CFPL (16.3 GPa). All these comparison differences (elongation, tensile strength and modulus) between the multi-CFPLs and unit-CFPL are attributed to the action of the second hot-press step. On the one hand, the multi-CFPLs become denser after the laminating-hot-press because some residual pores in its components (unit-CFPLs) were fully occupied. Therefore, the CF volume fractions of all multi-CFPLs were increased. On the other hand, the CF-PC interfacial bonding strength that was enhanced due to that additional holding time brought by the laminating-hot-press is beneficial to improve the impregnation process in porous material [23]. These two aspects are helpful to enhance tensile strength and modulus as well as reduce elongation.

Figure 7c–f show the fracture morphologies of multi-CFPLs with different layers of unit-CFPLs. All laminates show a relatively neat fracture section with only a little bit of pull-out CFs, which is similar to the unit-CFPL. No apparent difference in the fracture feature is observed between different laminates. This tensile appearance illustrates that with the second step in the forming process, the tensile properties of the unit-composite were well retained.

The flexural and tensile properties show stability in both performance and failure modes. The second step forming process, i.e., the laminating-hot-press, could realize a stable stack of laminates. The flexural strength of multi-CFPLs exceeds 300 MPa, and the modulus reaches 15 GPa, which reflects the good prospect of application for withstanding flexural loads as a beam structure [17,39,41].

### 3.5. Impact Property Analysis

A set of impact tests was conducted on all multi-CFPLs with unit-CFPL layers ranging from 1 to 9 prepared using the optimal laminating parameters discussed previously. Because its impact resistance is beyond 15 J, which is the maximum impact energy applied in this work, there is no discussion about the impact behavior of the 10-layer multi-CFPL.

**Figure 8:**
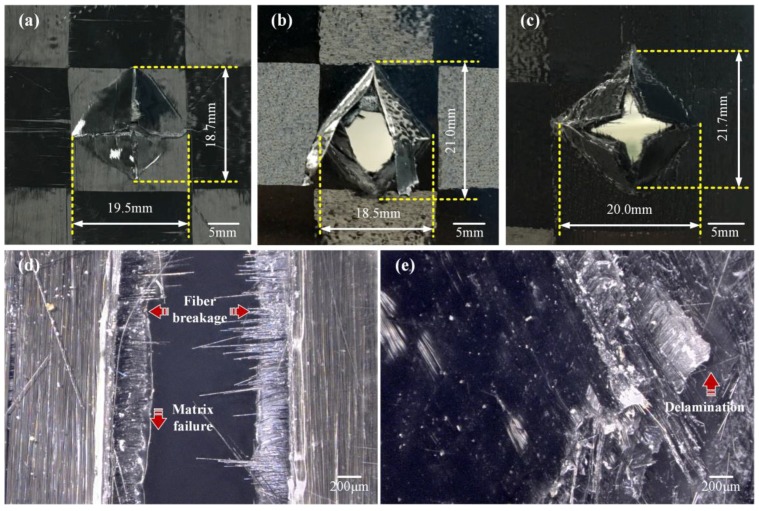
Impact fracture images of a multi-CFPL with different layer numbers of unit-CFPLs: (**a**) Unit-CFPL; (**b**) 3 layers; (**c**) 5 layers; (**d**,**e**) are the enlarged images of the unit-CFPL and 5-layer laminates, respectively.

Figure 8a–c show some typical impact fracture images captured on the multi-CFPLs with 1, 3 and 5 layers. They demonstrate typical four-sided pyramidal (tetrahedral) fractures, attributed to the bi-directional layup of the woven fabric composites [42]. All multi-CFPLs show a similar fracture morphology and exhibit a typical brittle fracture mode, which is definitely distinct from the plastic deformation of the PC sheet [23]. In addition, the fracture area of each laminate does not present an obvious variation trend with increasing layer numbers. The fracture area is limited by the unit cell area of weave carbon fabric, whose length equals the width of the pristine CF tow.

Figure 8d,e are two partial enlarged images taken of the fracture of the unit-CFPL and 5-layer multi-CFPL. The results indicate that there are three types of failure behaviors observed in these images: (i) Matrix failure, observed as the cracking of the matrix phase parallel to the fibers; (ii) delamination of the laminate layers due to interlaminar stresses; and (iii) fiber failure such as fiber breakage and fiber buckling. These characteristics have also been reported by Thanomsilp and Hog [43]. Compared with conventional composites [17,43], the matrix failure of the multi-CFPLs is much more apparent because of the relatively high matrix fraction, which achieves more than 80% in height. Moreover, the delamination defect of multi-CFPLs was found to increase as the growth of the unit-CFPL layer numbers. In addition, these laminates are also damaged due to the full penetration of the striker during impact tests.

**Figure 9:**
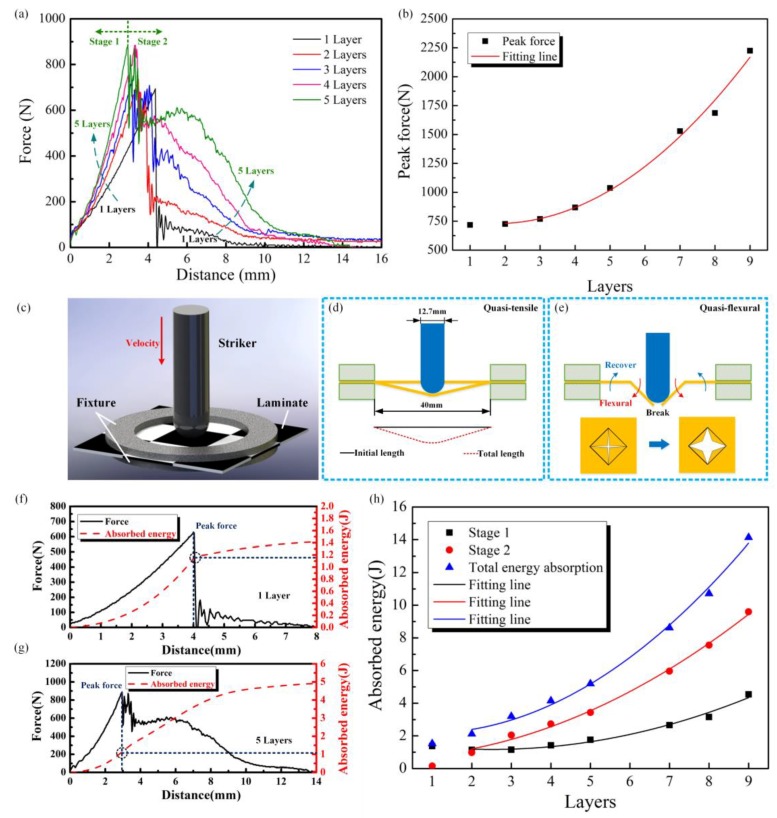
The impact properties of multi-CFPLs prepared by differently layered unit-CFPLs: (**a**) The force-distance curves of the multi-CFPLs with 1- to 5-layer unit-CFPLs; (**b**) the curve of the peak force versus layers; (**c**–**e**) are the schematic diagrams of the impact movement, a quasi-tensile stage and a quasi-flexural stage in an impact process; (**f**,**g**) are the energy absorption-distance and force-distance curves of the multi-CFPLs with 1-layer and 5-layer unit-CFPLs, respectively; (**h**) is the curves of energy absorption versus layers.

For a better understanding of the impact performance of multi-CFPLs, the impact distance-force data were recorded and analyzed, as shown in Figure 9a. For all multi-CFPLs, their impact forces first increase in an approximately linear tendency. After feeding a certain distance, these impact forces will achieve a peak value. At this moment, fiber failure is first observed, meaning that the peak value is the force limitation that a multi-CFPL can afford [44]. Moreover, the growth rate of the impact force seems to be proportional to the layer numbers of multi-CFPLs, i.e., the more unit-CFPL layers, the higher the growth rate of the impact force of multi-composites.

Furthermore, the impact distance of multi-CFPLs at the peak force moment is inversely proportional to the layer numbers of the unit-CFPLs. A thicker laminate always exhibits a shorter impact distance to achieve its maximum impact force. Figure 9b presents the peak force versus layers of multi-CFPLs. With polynomial fitting, the peak force and the layer display a two-polynomial growth relationship, whose degree of Adj. *R*-Square is 0.986, validating its good fitting quality, as listed in Table 1. Therefore, in terms of peak force, it is acceptable to design thick multi-CFPLs.

After reaching its peak value, the impact force will be sharply reduced by a certain value. Differing from the variation of peak force, this reducing value is inversely proportional to the layer number of the unit-CFPLs. The distance-force curve also indicates that a multi-CFPL with a larger layer number achieves its peak value in a shorter distance, whereas a longer distance is required to absorb all impact energy. According to the integral area of the distance-force curve, the entire absorbed impact energy of the multi-CFPLs increases with increasing unit-CFPL layer number.

To help in revealing the fundamental mechanism of this absorbed energy behavior, the whole penetration impact process of a multi-CFPL (shown in Figure 9c) is divided into two significant stages according to the peak force, as reported by Thanomsilp and Hogg [43], which is defined as quasi-tensile (happened first) and quasi-flexural (happened second) in this study. In this division, it is assumed that there is no fracture in the laminates before the impact force reaches its peak value, according to the failure mode proposed by Huber et al. [44]. These two stages were distinguished by the peak force on the distance-force curve as the boundary, and their schematic diagrams were exhibited in Figure 9d,e. The whole impact fracture process of the multi-CFPL is more similar to a combination of tensile and flexural behavior [9].

In the quasi-tensile stage, multi-CFPLs would extend under the compression of the striker and form a conic limited by the circle fixture. Along the fiber orientation, the deformation area could be regarded as a triangle. Meanwhile, the striker has a hemispherical shape. Therefore, the elongation zone of a laminate in two dimensions can be regarded as two parts—a straight line and an arc. In this work, the space diameter of the specimen fixture was 40 mm, and the impact displacement of the striker at the peak force moment of unit-CFPL was 4.36 mm. After a geometric calculation, the result shows that the calculated break elongation value is 2.40%, which is close to the tested break elongation (2.67%) of the unit-CFPL. The gap between these two values is probably caused by the neglected interlaminar shear stress in the pure tensile assumption because the interlaminar shear stress of the multi-CFPL must exist during the impact test.

In the quasi-flexural stage, the free sides in the failure area of the multi-CFPL were bent as the striker feeds. The impact force during this stage has a drastic force fluctuation that is similar to the final damage stage of the flexural stress-strain curve mentioned above. Since there are four free ends of the failure area, which means that there are four pieces occurring as different flexural deformations at the same time, the fluctuating frequency of the impact force in this stage is higher than the fluctuating frequency of the flexural tests mentioned above.

With the two-stage division of an impact process, the impact resistance capability of a multi-CFPL can also be divided into two corresponding parts [25]. Figure 9f,g show the force-distance and absorbed energy-distance curves of the multi-CFPLs with 1-layer and 5-layer unit-CFPLs, respectively. Obviously, the energy absorption proportion of the quasi-tensile stage and the quasi-flexural stage for multi-CFPLs differs as unit-CFPL layers change during the impact process. The total energy absorption in the quasi-tensile stage takes a major part for the one-layer multi-CFPL, with a proportion of more than 80%. By contrast, the energy absorption capacity for a five-layer multi-CFPL is dominated by the quasi-flexural stage, which occupies more than 70%. Traditionally, the peak force determines the energy absorption of conventional composites during complete penetration by the striker [43]. However, the total energy absorption capabilities of multi-CFPLs do not seem to be closely related to the peak force in this work. Although the peak force increases as the unit-CFPL layers increase, the rigidity of the multi-CFPLs also correspondingly increase, thereby reducing the impact distance, leading to the relatively slow energy absorption increase in the quasi-tensile stage. For the quasi-flexural stage, the bending resistance of multi-CFPLs increases as the unit-CFPL layers increase because of its enhanced rigidity, indicating an enhanced energy absorption capability in this stage.

Figure 9h illustrates the relationship between unit-CFPL layers and absorbed energies, including the energy absorbed in the quasi-tensile stage and in the quasi-flexural stage, and their summation. In addition, the scattered experimental data were fitted by polynomial lines, of which the specific fitting equations and the degree of the freedom-adjusted coefficient of determination (Adj. *R*-Square) are listed in Table 1. The one-layer multi-CFPL (unit-CFPL) is not considered in the fitting line because its CF-PC interfacial bonding strength is relatively low without the laminating-hot-press. The degrees of Adj. *R*-Squares of different laminates all exceed 0.97, validating the good fitting quality. These results indicate that the total energy absorption capabilities present a similar variation tendency and accelerated growth, which shows that the choice of thick laminates could obtain a higher impact performance than the choice of thin laminates. In addition, the increased rate of energy absorption in the quasi-flexural stage is much larger than the increased rate of energy absorption in the quasi-tensile stage. The absorbed energy in the quasi-flexural stage gradually dominates in the impact process as unit-CFPL layer numbers increase. In other words, the energy absorbing ability of a thick multi-CFPL mainly resists impact damage by its quasi-flexural process.

## 4. Conclusions

A multi-CFPL was manufactured by a novel two-step hot-press process based on the recyclability of thermoplastic PC. The optimal laminating parameters of this multi-CFPL in the laminating-hot-press were selected as 210 °C forming temperature, 6 MPa forming pressure, 3 min forming time and 10 min cooling time. Meanwhile, the unit-hot-presses were optimally prepared as 240 °C molding temperature, 6 MPa molding pressure and 2 min holding time.

The flexural strength of multi-CFPLs presents a slight declining tendency as the layers of the unit-CFPL increase, whereas the flexural modulus of a multi-CFPL is relatively stable. In addition, there is a unique point of first failure force in the flexural-strain curve, which can be used as an indication of the failure beginning in the first unit-CFPL layer during the flexural test. In addition, the tensile strength and modulus of the multi-CFPLs appear to have an approximately constant value of approximately 380 MPa and 17 GPa, respectively, which are slightly higher than the value of their pristine unit-CFPL. By contrast, the elongation of multi-CFPLs (2.50%) is slightly lower than the elongation of their pristine unit-CFPL (2.67%).

The impact failure behavior of multi-CFPLs demonstrates typical four-sided pyramidal (tetrahedral) fractures. There is a peak force in the impact force-distance, whose value is proportional to the layer numbers of multi-CFPLs, while the impact distance at the peak force moment is just the opposite. Moreover, the impact behavior of multi-CFPLs could be divided by peak force into two main stages—quasi-tensile and quasi-flexural processes. The absorbed energy in the quasi-flexural stage gradually dominates in the impact process as unit-CFPL layer numbers increase.

## Figures and Tables

**Table 1:** The fitting equations of peak force and absorbed energy for multi-CFPLs in the impact process, in which *x* represents the unit-CFPL layer numbers and *y* represents the objection.

Objection	Fitting Equation	Adj. *R*-Square
Peak force	*y* = 27.41823*x*^2^ − 96.38021*x* + 814.63241	0.98564
Absorbed energy in the quasi-tensile stage	*y* = 0.07501*x*^2^ − 0.37486*x* + 1.6387	0.97704
Absorbed energy in the quasi-flexural stage	*y* = 0.09994*x*^2^ + 0.07799*x* + 0.64861	0.99477
Total absorbed energy	*y* = 0.17494*x*^2^ − 0.29687*x* + 2.28731	0.99326
